# Heavy metal deposition and parameter change of soft contact lenses by exposure to particulate matter: Parameter change of SCL due to exposure to PM and heavy metal deposition

**DOI:** 10.1186/s12886-023-03154-2

**Published:** 2023-10-20

**Authors:** Won Young Jung, Jin Woo Kim, So Ra Kim, Mijung Park

**Affiliations:** 1 Department of Optometry, Seoul National University of Science and Technology, 232, Gongneung-ro, Nowon-gu, Seoul, Republic of Korea; 2 Bausch & Lomb, Seoul, Republic of Korea

**Keywords:** Air pollution, Particulate matter, Health effects, Heavy metals, Soft contact lenses

## Abstract

**Background:**

Particulate matter (PM) is known to contain heavy metals and be harmful to the tissues and organs of the human body including the eyes. As such, in this study, the deposition of heavy metals from PM on soft contact lenses was examined, and changes in the lens parameters were further investigated.

**Methods:**

Six types of soft contact lenses were exposed to captured PM_10_ for eight hours. The central thickness, water content, refractive power, and oxygen transmissibility of each contact lens were measured after analyzing the amounts of six heavy metals adsorbed on the contact lenses.

**Results:**

Lead, manganese, barium, arsenic, vanadium, and cadmium were detected in the captured PM, and only lead was adsorbed on all soft contact lenses except senofilcon C. The largest deposition was 23.21 ± 0.70 (10^− 3^)µg/lens of the lead on lotrafilcon B. The oxygen transmissibility of nelfilcon A exhibited statistically significant changes, however, it was within the ISO standard tolerance. Nevertheless, changes in the central thickness, water content, and refractive power of each soft contact lens were not statistically significant.

**Conclusions:**

This study revealed that a considerable amount of lead in PM_10_ was adsorbed on soft contact lenses. Amongst lens parameters, only oxygen transmissibility changed significantly. Thus, wearing soft contact lenses under high PM_10_ concentration might affect the physiology of the eyes.

## Background

Particulate matter (PM) is a liquid or solid matter with various chemical characteristics and sizes. PM has emerged as a global issue, and one of the reasons is because it was classified as a first-class carcinogen by the International Agency for Research on Cancer under the World Health Organization (WHO) in 2013 [1]. Most particulates are formed in the atmosphere as a result of complex reactions between chemicals, such as sulfur dioxide and nitrogen oxides that are pollutants emitted from power plants, industries, and automobiles [2]. Particles are defined by their diameter. Particles that are 2.5 microns or less in diameter are called PM_2.5_, those with a diameter of 10 microns or less are called PM_10_, and they can be inhaled deeply into the lungs [3].

Toxic heavy metals may be associated with PM, and such heavy metal components may vary depending on the surrounding environment [4, 5]. Also, heavy metals might react with oxygen and chloride in tissues and organs of the human body and exert their toxic effects [6, 7]. Essential elements to tissues and organs of the human body can be replaced with heavy metals, such as calcium with lead, zinc with cadmium, and most trace elements with aluminum [8, 9]. For example, zinc is a trace element and has a role in biological functions such as immune response, cellular proliferation and differentiation, growth and development, gene expression, and apoptosis; however, when the human body gets exposed to cadmium, zinc becomes the target of cadmium and cadmium binds to many bio-molecules. Thus, there’s a high chance that cadmium ion might amplify the biological signals and induce shift of the cellular redox balance [10]. Furthermore, PM may increase oxidative stress and free radicals by inducing antioxidant imbalances in tissues and organs of the human body, thereby damaging lipids, proteins, enzymes, and DNA [11].

The eyes are one of the few human organs that are continually exposed to the external environment, and are significantly affected by environmental factors such as temperature, humidity, wind, and lighting; PM could also be a factor that affects the human eyes [12]. Previous studies reported that an increase in the atmospheric PM concentration may increase the number of outpatients with conjunctivitis [13], and showed that PM may cause dry eye syndrome in model animals [14]. When soft contact lenses are worn, eyes can be subjected to the direct influence of the atmospheric environment as well as the indirect influence due to the changes in soft contact lenses caused by the atmospheric environment. As the parameters of soft contact lenses (such as wettability, water content, ionic properties, and oxygen transmissibility) may vary depending on the material characteristics and surface treatment, different lenses may have different changes in the same environment. For instance, larger aggregates of major tear proteins, lysozyme and albumin, were deposited on galyfilcon A and balafilcon A lenses compared to lotrafilcon A and B lenses. Also, more proteins in the form of larger aggregates were deposited on the polymacon material than on the silicone hydrogel material [15]. The degree of protein deposition on soft contact lenses may vary depending on the bond between the surface charge and the protein charge. Cationic proteins are more easily attached to anionic material lenses than to cationic or neutral materials [16].

Previous studies indicate that the adhesion of PM_2.5_ components (including heavy metals) may vary between rigid gas permeable and soft contact lenses [17]. In this case, there is a possibility that deposition of PM components on soft contact lenses, might cause changes in surface characteristics and water content, and such changes may induce parameter changes that affect the wearing comfort or vision correction effect of contact lenses, such as central thickness and refractive power. Moreover, when contact lenses act as the storage of heavy metals due to the deposition of heavy metals from PM, they might have an adverse effect on eye health. To address these problems, it is necessary to conduct research into PM influence on contact lenses. As such, in this study, the deposition of heavy metals from PM_10_ on soft lenses and the amount of deposition according to the lens material were investigated. In addition, changes in the parameters of the soft lenses exposed to PM_10_ were evaluated and compared according to the material characteristics.

## Methods

All soft lenses were deionized in distilled water for 48 h before being exposed to PM_10_, in order to minimize the effect of packing solution. A total of 6 lenses were respectively used for the component analysis and parameter measurement in the experimental and control groups of each material. All measurements were triplicated with three separate sets. Soft lenses in the PM_10_ exposure group were exposed to a PM_10_ solution for 8 h and then their surfaces were agitated with distilled water for 40 min. Soft lenses in the control group were treated in the same way in a solution without PM_10_. Soft lenses for component analysis were stored in distilled water by further analysis. Only for parameter measurement, the lenses were immersed in phosphate-buffered saline (PBS) solution of the ISO 18369-3 standard for over 24 h prior to measurement.

### Soft contact lenses targeted

The target soft lenses included two types of hydrogel lenses and four types of silicone hydrogel lenses with different monomer types used for synthesis or different wetting agent contents (Table 1).

**Table 1:** Specification of soft contact lenses used in this study

Classification	Hydrogel lens		Silicone hydrogel lens			
Brand name	FOCUS DAILIES	DAILIES AquaComfort Plus	ACUVUE Vita	Air Optix Aqua	PureVision2 HD	Biofinity
USAN^*^	Nelfilcon A	Nelfilcon A^°^	Senofilcon C	Lotrafilcon B	Balafilcon A	Comfilcon A
Polymer	HEMA + PVA	HEMA + PVA, HPMC, PEG	mPDMS + DMA + HEMA + TEGDMA + PVP + SiGMA	DMA + TRIS + siloxane monomer	NVP + TPVC + NCVE + PBVC	FM0411M; HOB; IBM; M3U; NVP; TAIC; VMA
FDA Group	II	II	5Cr	5Cm	5A	5C
Wearing modality	Daily	Daily	Monthly	Monthly	Monthly	Monthly
Manufacturer	Alcon	Alcon	Johnson & Johnson	Alcon	Bausch & Lomb	Coopervision
Diameter(mm)	13.8^a^	14^a^	14^a^	14.2^a^	14^a^	14^a^
Thickness at -3.00 D(mm)	0.1^a^	0.1^a^	0.07^a^	0.08^a^	0.07^a^	0.08^a^
Base curve(mm)	8.6^a^	8.7^a^	8.4/ 8.8^a^	8.6 ^a^	8.6^a^	8.6^a^
Visible light transmission(%)	96^b^	≥ 92^b^	89 to 99^b^	≥ 96^a^	≥ 95^a^	≥ 97^a^
Water content(%)	69^a^	69^a^	41^a^	33^a^	36^a^	48^a^
Oxygen Permeability, Dk (10^− 11^)^‡^	26^a^	26^a^	103^b^	110^a^	91^b^	128^a^
Oxygen transmissibility, Dk/t (10^− 9^)^†^	26^a^	26^a^	147^a^	138^a^	130^a^	160^a^
Surface treatment	None	None	None	Plasma polymerization	Plasma oxidation	None

^*^ United States Adopted Name

^‡^ Oxygen permeability; Dk: (cm^2^/sec)(mL O_2_/mL * mmHg)

^†^ Oxygen transmissibility; Dk/t: (cm /sec)(mL O_2_/mL * mmHg)

^°^ HEMA + PVA, HPMC, PEG

^a^ based on available public information and manufacturer measurements

^b^ based on available public information and FDA measurements

HEMA: 2-hydroxyethyl methacrylate; MA: methacrylic acid; PVP: polyvinylpyrrolidone; PVA: polyvinyl alcohol; HPMC: hydroxypropyl methylcellulose; PEG: polyethylene glycol; DMA: N,N-dimethylacrylamide; TRIS: trimethylsiloxy silane; NVP: N-vinyl pyrrolidone; TPVC: tris-(trimethylsiloxysilyl)propylvinyl carbamate; NCVE: N-carboxyvinyl ester; PBVC: poly(dimethylsiloxy)di(silybutanol)bis(vinyl carbamate); FM0411M, α-methacryloyloxyethyl iminocarboxyethyloxypropyl-poly(dimethylsiloxy)-butyldimethylsilane; HOB: 2-hydroxybutyl methacrylate; IBM: isobornyl methacrylate; M3U: αω -bis(methacryloyloxyethyl iminocarboxy ethyloxypropyl)-poly(dimethylsiloxane)-poly(trifluoropropylmethylsiloxane)-poly(ω-methoxy-poly(ethyleneglycol)propylmethyl-siloxane; TAIC: 1,3,5-triallyl-1,3,5-triazine-2,4,6(1H,3H,5H)-trione; VMA:N-Vinyl-N-methylacetamide.

### PM_10_ collection and measurement

The PM_10_ was collected using a high-volume air sampler on the rooftop of Cheongwoongwan Building, Seoul National University of Science and Technology, which is located in Nowon-gu, Seoul, South Korea, for 24 days (585 h and 34 min) without rain between October 27th and December 8th in 2017. Collecting period was chosen due to the cool weather, because air pollutions are severe in the cool seasons owing to dry condition. The suction flow rate was 5.0 L/min, and three QM-A quartz microfiber filters were used. Based on official data from the Nowon-gu monitoring station of the Ministry of Environment of South Korea on the days of PM_10_ collection, the mean PM_10_ concentration (based on 24 h) was 47.47 ± 14.06 µg/m^3^, which met the WHO PM_10_ (particulate matter with an aerodynamic diameter less than 10 µm) average guideline of 50 µg/m^3^ based on 24 h. Upon completion of PM_10_ collection, QM-A quartz microfiber filter was put on electronic scale (PAG214C, Ohaus, USA) and measured in 0.001 g. The value of the weight difference before and after PM_10_ collection was calculated as the amount of PM_10_.

The three filters were stirred in 200 mL of distilled water each, for 24 h. This process was repeated five times with 20 min of sonication followed by a 5-minute break to extract the PM_10_ particles and obtain the first PM_10_ solution with a volume of 600 mL. Subsequently, each filter was subjected to a second extraction of the PM_10_ particles, resulting in 100 mL of PM_10_ solution from each filter. Overall, this process yielded a total volume of 300 mL of PM_10_ solution. For the second extraction, only 300 mL of distilled water was used instead of 600 mL as in the first extraction, in order to avoid unnecessary dilution of the solution. Finally, the first and second solutions were mixed to produce a final PM_10_ solution with a total volume of 900 mL. Each soft lens was exposed to the 1.5 mL PM_10_ solution for 8 h. The amounts of heavy metals contained in the PM_10_ solution was measured using inductively coupled plasma-mass spectrometer (ICP-MS) (iCAP-Q, Thermo, Germany). The soft lens was pretreated using 5 mL of nitric acid (69%, Wako, Japan) and a microwave digestion system (Multiwave7000, Anton Paar, Austria), and the experiment was conducted after correcting the concentration of the element detected by blank analysis.

The concentration of heavy metal in the PM_10_ solution was measured in parts per billion (ppb), converted into µg/m^3^, and calculated proportionally to the amount of the divided solution (1.5 mL) to expose the soft lens to PM_10_.

### Central thickness and refractive power measurement

The central thickness of each soft lens was measured using an electronic thickness gauge (ET-3, Createch, USA), and an automatic lensmeter (CL-300, Topcon, Japan) was used to measure refractive power.

### Water content measurement

Each soft lens was weighed to 0.001 g prior to drying and was weighed again after being dried in a vacuum oven at 65℃ for more than 24 h until a stable weight was achieved. The two weights were applied to the following equation.

$$ Water\, content\left(\%\right)=\frac{{m}_{1}-{m}_{2}}{{m}_{1}}\times 100$$

(m_1_: Soft lens weight before drying, m_2_: Soft lens weight after drying)

### Oxygen transmissibility measurement

Oxygen transmissibility of each soft lens was measured based on the polarographic method of ISO 18369-4:2017. The device used in the experiment was an O_2_ Permeometer (201T, Createch, USA), and the measurement was performed at 95.00% humidity and 35 ± 0.5°C using a constant temperature and humidity chamber (Daihan, WTH-E 155, Korea).

### Statistical analysis

Results were expressed as the mean ± standard deviation, and statistical analyses were conducted using SPSS 18.0 for Windows. The statistical significance of changes in the parameters of the soft lenses exposed to PM_10_ compared with the control group, and differences in the amount of heavy metal deposition between the soft lenses exposed to PM_10_, was verified by the Mann-Whitney U test. If the *p* value was 0.05 or less, it was determined to be statistically significant.

## Results

### Heavy metal concentrations in the atmosphere

Six heavy metals were detected in the PM_10_ solution. Lead, manganese, and barium concentrations were > 200 (10^3^)µg/m^3^, whereas those of arsenic, vanadium, and cadmium were < 90 (10^3^)µg/m^3^. Mercury and beryllium were not detected (Table 2).

**Table 2:** Heavy metal concentration in PM_10_ solution

Heavy metal	Concentration[(10^3^)µg/m^3^] (%)
Pb	281.21 ± 10.94 (28.8)
Mn	277.85 ± 27.70 (28.4)
Ba	225.85 ± 6.22 (23.1)
As	89.97 ± 5.60 (9.2)
V	76.28 ± 3.88 (7.8)
Cd	26.60 ± 0.33 (2.7)

Values were expressed as MEAN ± SD

### PM_10_ deposition on soft contact lenses according to the lens material

Heavy metal deposition on soft lenses that were exposed to the PM_10_ solution was examined. It was found that lead, manganese, and barium were deposited, whereas arsenic, vanadium and cadmium were not (Table 3). Manganese and barium were deposited only on lotrafilcon B, lead was deposited on all soft lenses except for senofilcon C.

**Table 3:** Amount of heavy metal deposition of soft contact lens

Contact lens	Heavy metal deposition, (10^− 3^)µg/lens					
	Pb	Mn	Ba	As	V	Cd
Nelfilcon A	8.67 ± 1.79	0	0	0	0	0
Nelfilcon A^°^	17.51 ± 2.80	0	0	0	0	0
Senofilcon C	0	0	0	0	0	0
Balafilcon A	6.63 ± 1.14	0	0	0	0	0
Lotrafilcon B	23.21 ± 0.70	9.19 ± 0.39	8.92 ± 0.58	0	0	0
Comfilcon A	5.22 ± 0.12	0	0	0	0	0

Values were expressed as MEAN ± SD.

### Central thickness

Hydrogel lenses were more affected by PM_10_ exposure than silicone hydrogel lenses. There was, however, no statistically significant change in the central thickness for the control group or the PM_10_ exposure group (Table 4).

**Table 4:** The comparison of central thickness, refractive power, and oxygen transmissibility between control group and PM_10_ exposure group

	Contact lens	Control	PM_10_ exposure	Relative ratio(%)	p-Value
Central thickness(mm)	Nelfilcon A	0.095 ± 0.004	0.093 ± 0.005	97.89	0.753
	Nelfilcon A^°^	0.092 ± 0.003	0.092 ± 0.002	100.00	0.743
	Senofilcon C	0.072 ± 0.002	0.072 ± 0.002	100.00	0.750
	Balafilcon A	0.077 ± 0.005	0.078 ± 0.002	101.30	0.281
	Lotrafilcon B	0.071 ± 0.004	0.071 ± 0.003	100.00	1.000
	Comfilcon A	0.067 ± 0.003	0.067 ± 0.006	100.00	0.753
Refractive power(D)	Nelfilcon A	-2.91 ± 0.17	-2.94 ± 0.13	101.03	0.117
	Nelfilcon A^°^	-3.02 ± 0.16	-3.04 ± 0.08	100.66	0.465
	Senofilcon C	-2.87 ± 0.13	-2.87 ± 0.05	100.00	0.295
	Balafilcon A	-2.92 ± 0.25	-3.04 ± 0.21	104.11	0.675
	Lotrafilcon B	-2.99 ± 0.07	-3.02 ± 0.25	101.00	0.917
	Comfilcon A	-2.82 ± 0.08	-2.80 ± 0.06	99.29	0.599
Oxygen transmissibility (10^− 9^) ^†^	Nelfilcon A	24.46 ± 0.15	27.13 ± 0.95	110.92	0.049^*^
	Nelfilcon A^°^	26.58 ± 0.44	28.79 ± 0.74	108.31	0.049^*^
	Senofilcon C	111.73 ± 11.80	114.00 ± 8.27	102.03	0.827
	Balafilcon A	100.89 ± 3.76	99.31 ± 7.41	98.43	0.827
	Lotrafilcon B	101.30 ± 3.52	100.49 ± 8.53	99.20	0.827
	Comfilcon A	114.94 ± 3.86	114.03 ± 5.70	99.21	0.827

^†^ (cm /sec)(mL O_2_/mL * mmHg)

Values were expressed as MEAN ± SD

^*^ Significantly different at p 0.05 by Mann-Whitney U test

### Water content

The change in the water content of the PM_10_ exposure group compared to the control group was not statistically significant (data not shown).

### Refractive power

For the PM_10_ exposure group, the refractive power showed a tendency to increase compared to the control group, however, the refractive power change in all soft lenses was not statistically significant (Table 4).

### Oxygen transmissibility

Oxygen transmissibility change rates of all the soft lenses in the control group and the PM_10_ exposure group were less than 15.00% (Table 4). Oxygen transmissibility of the PM_10_ exposure group compared to the control group decreased for many of the silicone hydrogel lenses, whereas it increased for many of the hydrogel lenses. Statistically significant differences were observed only in the hydrogel lenses. Changes in the oxygen transmissibility of the hydrogel lenses, however, were within the ISO standard tolerance.

## Discussion

Heavy metal concentrations in the atmosphere are affected by the geographic environment, season, and temperature [18]. Such concentrations may vary depending on the region even in the same country [19]. Heavy metal components that were measured in this study, however, are also present in the PM_10_ of other urban areas [19, 20]. Therefore, the results of this study can be applied to other areas.

Among the tested soft lenses, lotrafilcon B that was manufactured from low-water-content ionic materials exhibited the largest heavy metal deposition, than nelfilcon A and nelfilcon A^°^ (high-water-content non-ionic materials), followed by balafilcon A, comfilcon A, and senofilcon C (low-water-content non-ionic materials). Among the soft lens material characteristics, the water content and ionic property are known to significantly affect the amount of tear protein deposition and so does the temperature caused by the surrounding environment [16, 21–22]. According to previous study, low water non-ionic lenses take up the lowest quantity of tear protein and on the other hand, high water ionic lenses take up the highest quantity of tear protein [23]. Similar to protein deposition aspect, in our study, heavy metal deposition showed the highest in lotrafilcon B (low-water-content ionic material), and the lowest in balafilcon A, comfilcon A, and senofilcon C (low-water-content non-ionic materials). Therefore, it is suggested that heavy metal deposition tendencies are closely related with tear protein deposition of soft contact lenses. It is also expected that the electrostatic bond due to the attraction between cations and anions can affect the amount of heavy metal deposition. This may explain why senofilcon C exhibited the lowest level of heavy metal deposition; however, further investigation is required to provide a comprehensive understanding of this phenomenon.

In this study, there was no relationship between the atomic weights of heavy metals and the amount of soft lens deposition. In the case of ions, however, heavy metals with a + 2 oxidation state(Pb, Mn, Ba, V, Cd), as well known [24–26], showed a tendency to bond well with the lenses. The ionic property of the soft lenses occurred because methacrylic acid was included in the monomers to be copolymerized. Further work is required to assess the connection between contact lens ionic properties and heavy metals in PM_10_.

Lead was deposited on almost all soft lenses, while manganese and barium were deposited on some soft lenses. Lead, which was deposited on the largest number of lenses in this study, has been known to cause oxidative stress, which occurs due to an increase in reactive oxygen species and toxicity. Oxidative stress in the eyes may cause various forms of cell damage, such as protein oxidation, DNA destruction, apoptosis, and lipid peroxidation [27]. If eye tissues are continuously exposed to oxidative stress, it may cause cataract [28].

The WHO air quality standard of lead is < 0.5 µg/m^3^, based on the annual mean. The experiment was designed to mimic the exposure of soft lenses to PM_10_ in the atmosphere. However, further research is needed to investigate the direct impact of air quality, particularly on days with severe PM_10_ condition, on soft lenses. If a simple comparison is performed using only the values, however, the lead concentration in the PM_10_ solution was 281.21 ± 10.94 (10^3^)µg/m^3^, and 23.21 ± 0.70 (10^− 3^)µg/lens of lead concentration was deposited on lotrafilcon B, which was the largest amount of lead deposition. The result was lower than the air quality standard. The WHO air quality standard of manganese (which causes neurotoxicity) is 0.34 µg/m^3^ based on the annual mean [29]. Its deposition amount on lotrafilcon B in this study was 9.19 ± 0.39 (10^− 3^)µg/lens, which was also lower than the air quality standard. In the case of barium (which causes neurotoxicity and myotoxicity) [30], the drinking water standard of 7.30 µg/m^3^ was used because a WHO air quality standard has not been established. The barium deposition amount on lotrafilcon B, which exhibited the largest barium deposition, was 8.92 ± 0.58 (10^− 3^)µg/lens. Heavy metal deposition amounts on soft lenses in an actual environment, however, may differ because the PM_10_ solution used in this study contained PM_10_ collected for approximately 1.15 h.

The parameter change of the PM_10_ exposure group was observed in oxygen transmissibility. There was no statistically significant change in the central thickness, which is related to the wearing comfort and strength of a contact lens, and the refractive power, which is a main optical characteristic. The oxygen transmissibility change was statistically significant only for the PM_10_ exposure group among the hydrogel lenses (nelfilcon A and nelfilcon A^°^), but not for the silicone hydrogel lenses (senofilcon C, balafilcon A, lotrafilcon B, comfilcon A). For hydrogel lenses, the oxygen transmissibility generally increased as the water content increased and the thickness decreased, because oxygen is transferred to the cornea through water. In the case of the thickness, which is calculated as a single value in the denominator, there was no difference between the control group and the PM_10_ exposure group, indicating that the oxygen transmissibility change was not caused by the thickness, but oxygen permeability. Some lenses, however, exhibited a statistically significant increase in oxygen transmissibility. These lenses were hydrogel lenses, for which the increase or decrease in water content directly and significantly affected oxygen permeability usually, but in this study, there were no significant changes in water content. This indicates that PM_10_ components caused changes that cannot be explained only by the quantitative value referred to as the water content. The water present in a soft lens is bound water combined with the lens material and free water that moves freely in the lens. For the water content, both types of water are measured. Therefore, the proportions of free water and bound water can be changed by PM_10_ components, and this change may have affected oxygen diffusion and dissolution [31]. In other words, HEMA (Hydroxyethyl methacrylate), which is the basic monomer of a hydrogel lens, has hydroxyl groups composed of hydrogen and oxygen at the end of the molecular structure, and it is possible that these hydroxyl groups formed new bonds by adsorbing heavy metal cations and affected the amount of free water.

In addition to the above changes in the contact lens parameters, even a small change on the contact lens surface caused by PM_10_ components may affect eye-related symptoms that cause changes in wearing comfort. Examples can be found in studies that report on changes in the characteristics of a contact lens affect the interaction among lipids, proteins, mucins, and electrolytes, which are tear film components [32]. Besides, changed deposition and denaturation of each component may stimulate the immune response of the eyes or cause osmotic changes [33–36].

In this study, all measurements were obtained after exposing contact lenses to the PM_10_ solution for 8 h. We acknowledge the presence of several limitations in this study. Firstly, due to its in vitro design, it is challenging to ascertain the potential impact of heavy metal deposition from particulate matter when wearing contact lenses on days with elevated air pollution levels, and its consequences on ocular health and the human body. While existing literature suggests adverse effects of heavy metals on human health, the specific implications through contact lens usage remain unexplored. Secondly, a comprehensive and detailed analysis of the lens polymers utilized in this study and their direct association with the deposition of heavy metals from particulate matter prove to be intricate. Therefore, further research is needed to explore these aspects and obtain a better understanding. Also, further studies on the effects of repeated or long-term exposures are required. In fact, the replacement of lenses at longer intervals than the specified period was reported as the most common risk behavior among the contact lens users [37]. In other words, the results of the eight-hour exposure may not fully reflect the daily lens wearing time in everyday life. Even though contact lenses were exposed to the PM_10_ solution for 8 h, lotrafilcon B (monthly-wear lens) had the largest amount of heavy metal deposition. If these monthly-wear lenses are exposed to heavy metal repeatedly or longer, there might be much more heavy metal deposition and the soft lens parameters change due to the deposition of heavy metals from PM_10_ may also lead to results different from those of this study.

## Conclusions

The result of this study revealed that the amount of heavy metal deposition varies depending on the material of soft contact lenses, and different lens materials exhibit distinct patterns of changes in lens parameters following exposure to heavy metals. Based on these findings, the oxygen transmissibility of nelfilcon A and nelfilcon A^°^ contact lenses in the exposure group was significantly lower than that of lenses made from the same material in the control group. Thus, further study is needed to investigate the potential impact on eye health. Therefore, research on the accumulation of PM_10_ components needs to be conducted for different soft lenses modalities.

## Data Availability

The data that support the findings of this study are available from the corresponding author upon reasonable request.

## Abbreviations

PM: Particulate matter
WHO: World Health Organization
PBS: Phosphate buffered saline
ICP-MS: Inductively coupled plasma-mass spectrometry
